# Adaptive Peak Tracking as Explanation of Sparse Fossil Data Across Fluctuating Ancient Environments

**DOI:** 10.1002/ece3.71705

**Published:** 2025-07-03

**Authors:** Rolf Ergon

**Affiliations:** ^1^ University of South‐Eastern Norway Norway

**Keywords:** adaptive landscape, adaptive peak tracking, AIC, bryozoans, fitness landscape, fossil record, optimal phenotypic values

## Abstract

Species that have persisted over millions of years have done so because they have been able to track peaks in an adaptive landscape well enough to survive and reproduce. Such optima are defined by the mean phenotypic values that maximize population mean fitness, and they are predominantly functions of the environment, for example the sea temperature. The mean phenotypic values over time will thus predominantly be determined by the environment over time, and the trait history may be found in the fossil record. Here, I simulate such a tracking system, using both a basic non‐plastic selection model and a univariate intercept‐slope reaction norm model. I show how both linear and nonlinear mean phenotype versus environment functions can be found also from quite sparse and short time series from the fossil record, and I discuss how this methodology can be extended to multivariate systems. The simulations include cases with a constraint on the individual trait values and with other factors than environment influencing the position of the adaptive peak. The methodology is finally applied on a time series of mean phenotypic values in a record of *Microporella agonistes* bryozoan fossils spanning 2.3 million years, using the $\partial^{18}O$ measure as proxy for sea water temperature. From as few as nine samples of mean phenotypic values found in the fossil record it was possible to identify a linear mean phenotype versus environment function by means of the weighted lest squares (WLS) method, and to predict the continuous mean phenotypic values as functions of time with prediction errors within or just outside the standard errors of the observations. Leave‐one‐out cross validation gave satisfactory results. The Akaike Information Criterion (AIC) shows that the WLS model outperforms alternative random walk models. It remains to verify predictions for longer time periods without known or investigated fossil data.

## Graphical abstract

Species that have persisted over millions of years have done so because they have been able to track peaks in an adaptive landscape. Here, I simulate such a tracking system, using both a basic non‐plastic selection model and a univariate intercept‐slope reaction norm model. The methodology is finally applied on a time series of mean phenotypic values in a record of bryozoan fossils spanning 2.3 million years, and based on nine samples it was possible to predict the continuous mean phenotypic values as functions of time with prediction errors within or just outside the standard errors of the observations.

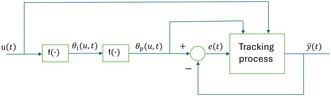

## 1 Introduction

Evolutionary time series in the fossil record provide information on phenotypic changes over millions of years. Such time series may consist of quite sparse samples, and they are traditionally modeled by various forms of random walk processes (Hunt 2006; Voje 2023), or by Ornstein‐Uhlenbeck (OU) processes describing evolution of mean phenotypic values toward fixed or moving peaks in the adaptive landscape (Voje 2023). Here, the adaptive landscape and adaptive peak metaphors assume that the population mean fitness is a function of possibly multivariate mean phenotypic values; see Pigliucci (2013) for the historical background and a clarifying discussion.

As an alternative to the traditional fossil time series modeling, I propose that adaptive peak tracking may be a useful modeling approach, and that this requires two types of landscape metaphors. First, I assume a fitness landscape where individual fitness is a function of a possibly multivariate set of individual phenotypic values. Second, I assume an adaptive landscape where the population mean fitness is a function of a possibly multivariate set of mean phenotypic values. For simplicity, I assume that the position of the individual fitness peak is a function of a dominating and well‐known environmental factor, from which follows that the adaptive peak also is a function of this environmental factor, but that it may have a somewhat different position owing to non‐symmetrical individual fitness or trait distribution functions. I am thus not attempting to estimate the effect of various selective or other factors on the position of adaptive optima (Hansen 1997), but I just assume that various selective factors over time keep the mean phenotypic values in the vicinity of the adaptive peak, and as an example, I will use the relative size of feeding zooids (autozooids) in encrusting cheilostome bryozoans. Liow et al. (2017) studied communities of such organisms spanning more than 2 million years, and an interesting result was that six different species showed temporally coordinated changes in average zooid sizes, suggesting that the different species responded to a common external driver. Liow et al. (2017) and Liow et al. (2024) pointed to sea temperature as a potential common driver, but they found that the available time series of changes in mean zooid size lacked sufficient length to rigorously test such a hypothesis. In my view, a study by means of an adaptive peak tracking model, as introduced above, is clarifying.

**FIGURE 1:**
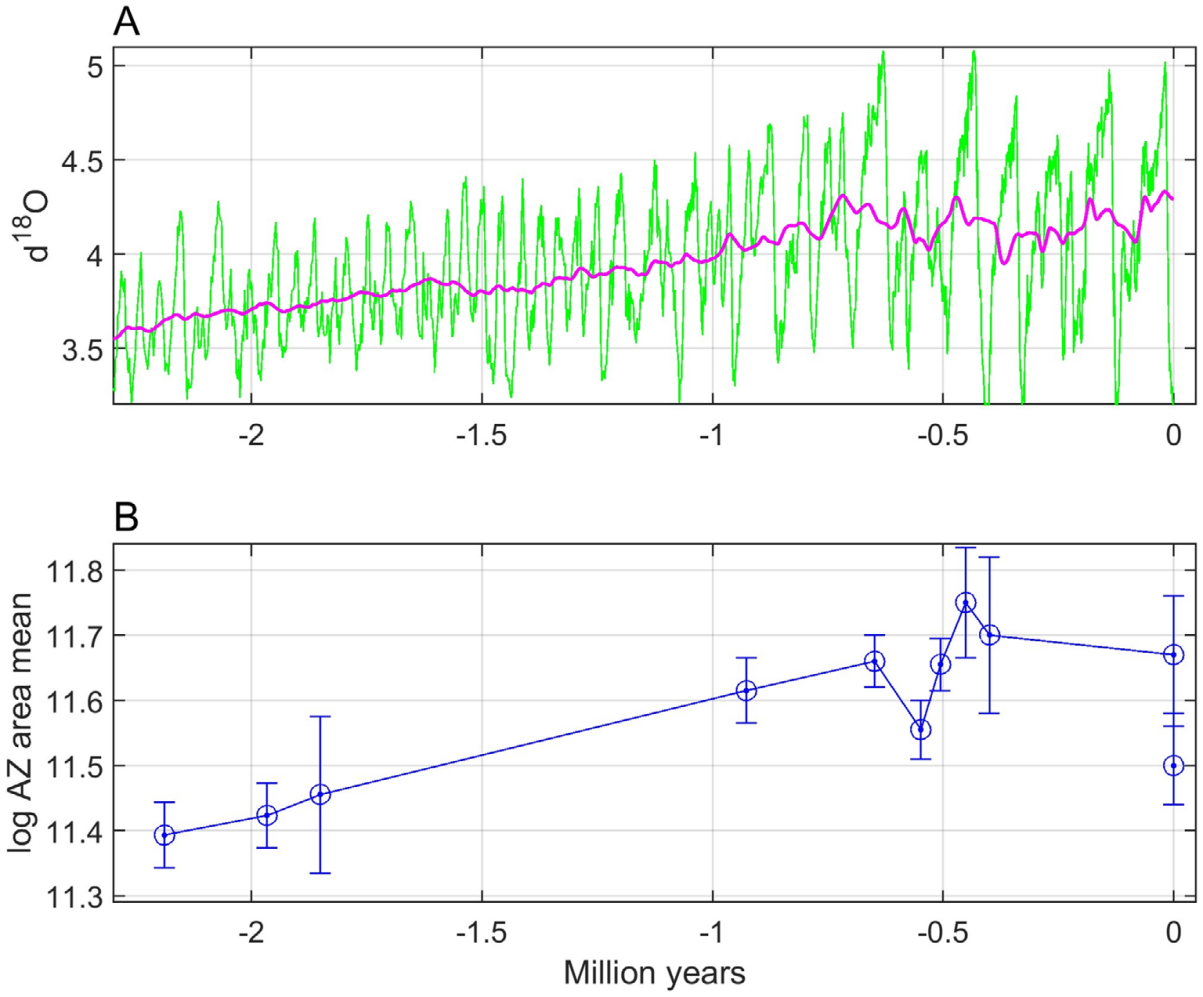
Raw $u\left(t\right)=\partial^{18}O\left(t\right)$ data (panel A, green line) and a centered moving average signal $u_{\text{filt}}\left(t\right)$ computed from a sliding window of length 100 samples along the time axis (panel A, red line, see Section 4 for details), as well as *log AZ area mean* (panel B, circles and dots) with standard errors as in figure S2 in Liow et al. (2024). Present time is used as zero point.

For an introduction, Figure 1 shows data from Liow et al. (2024) (see a more detailed presentation in Section 4), where the $\partial^{18}O\left(t\right)$ measure is a widely used proxy for seawater temperature (Lisiecki and Raymo 2005). A natural question is now which type of model we need for explanation of the *log AZ area mean* data in panel B given the $\partial^{18}O\left(t\right)$ data in panel A, and as already mentioned an adaptive peak tracking model is in my view a viable alternative.

**FIGURE 2:**
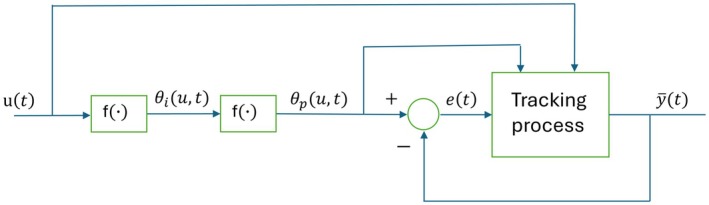
Block diagram for adaptive peak tracking system, with the environmental variable $u\left(t\right)$ as input and the population mean phenotypic value $\bar{y}\left(t\right)$ as output. The phenotypic values $\theta_{i}\left(u,t\right)$ and $\theta_{p}\left(u,t\right)$ that maximize individual and population mean fitness, respectively, are together with the tracking error $e\left(t\right)$ internal signals in the system. See text for explanations of blocks marked by $f\left(∙\right)$ symbols.

A block diagram for the proposed adaptive peak tracking model is shown in Figure 2, and as described above it is based on two types of landscape metaphors. The optimum phenotype that maximizes individual fitness is assumed to be a function $\theta_{i}\left(u,t\right)=f_{i}\left(u\left(t\right)\right)$, where, $u\left(t\right)$ is the dominating and well‐known environmental variable (in the bryozoan example the $\partial^{18}O\left(t\right)$ measure). The optimum mean phenotypic value that maximizes population mean fitness is assumed to be a function $\theta_{p}\left(u,t\right)=f_{p}\left(u\left(t\right)\right)$, deviating from $\theta_{i}\left(u,t\right)$ when either the individual fitness function or the trait distribution is non‐symmetrical. When both the fitness function and the trait distribution are symmetrical, we will thus have $\theta_{p}\left(u,t\right)=\theta_{i}\left(u,t\right)$ (see Section 2 for an example with Gaussian functions). The tracking process in Figure 2 is attempting to keep the mean phenotypic value $\bar{y}\left(t\right)$ (in the bryozoan example the *log AZ area mean* value) in the vicinity of the optimal value $\theta_{p}\left(u,t\right)$, such that the tracking error $e\left(t\right)$ is kept small. The basic assumption is thus that the tracking process keeps the tracking error small, regardless of the details of this process.

A fluctuating environment as shown in Figure 1 corresponds to an adaptive landscape where the adaptive peak position fluctuates, and how well the peak is tracked depends on the properties of the evolutionary system. For a population with phenotypic plasticity, the tracking error may be kept small at all times, while a population without plasticity may only be able to track a moving average as indicated in Figure 1. However, a population without plasticity may also keep the tracking error small, provided that the additive genetic variances are large. Sparse fossil data will in any case not be informative enough for predictions of strongly fluctuating mean phenotypic values, and a remaining possibility is therefore to predict moving averages over a window size that may be used as a prediction parameter. In cases where the fossil phenotypic data are mean values from given time periods, a natural choice is also to use mean environmental data from these time periods as input to the tracking model (see Section 4 for an example).

The assumption that the mean phenotypic value stays in the vicinity of the adaptive peak is supported by findings regarding multiple species and traits indicating that “fossil lineages have ascended adaptive peaks and remained at their summits as they shift position through time” (Bell 2013). An important reference is here Estes and Arnold (2007), who based on a large data set found that “the underlying process causing phenotypic stasis is adaptation to an optimum that moves within an adaptive zone with stable boundaries,” which applied to the data in Figure 1B, and considering the standard errors given, means that *log AZ area mean* moves between approximately 11 and 12. Using a database over many contemporary populations, Estes and Arnold (2007) also found that phenotypic means are typically close to the adaptive peak (46% within 1 phenotypic standard deviation of the optimum, and 65% within 2 standard deviations). Estes and Arnold (2007) based their results on tests with a single displacement of the optimum, using an extensive set of data (Gingerich 2001), but they acknowledged that the actual optimum might move many times with a small total net displacement. That is a good description of the optimal mean phenotype corresponding to the environmental data in Figure 1A, with large short‐term variations and a rather limited long‐time net variation. I thus assume that short‐term environmental fluctuations as in, for example, Figure 1A, result in variations in optima within a stable adaptive zone (Uyeda et al. 2011). However, contrary to Uyeda et al. (2011) I assume a causal tracking system and not a purely stochastic model. In real cases there will of course be stochastic variations in the environmental variable and in the position of the individual fitness peak, and additional stochastic factors that force the adaptive peak to deviate from the individual fitness peak. Such stochastic variations are included in simulations, but further discussion of this topic is beyond the scope of this paper.

A persistent system excitation in the form of short‐term environmental fluctuations, as exemplified in Figure 1A, makes it unlikely that a tracking system, as shown in Figure 2, gets stuck in a local optimum or in a valley in the adaptive landscape. As illustrated in simulations, it is more likely that the mean phenotypic values fluctuate around global optima; see, however, an example of slow adaptation in Section 3.5 and a discussion in Section 5.

In simulations I assume that the tracking process block in Figure 2 can be described by use of Lande's (1979) selection theory for populations without plasticity (Lande 1979), which was also used by Estes and Arnold (2007), but I also include examples with phenotypic plasticity (Lande 2009). In real cases, however, other mechanisms such as, for example, adaptive walks in response to mutations may also be involved (Walsh and Lynch 2018, Ch. 27). It is as already mentioned essential to realize that although knowledge of the details of the tracking process may be interesting and valuable, such knowledge is not necessarily needed for finding continuous predictions $\hat{\bar{y}}\left(t\right)$ based on sparse measurements of mean phenotypic values $\bar{y}\left(t\right)$.

Under the assumption that the mean trait value has remained close to the adaptive peak as it has shifted position through time, such that the following error $e\left(t\right)$ in Figure 2 has remained small relative to the width of the underlying mean fitness function, the position of the adaptive peak as function of the environment, $\theta_{p}\left(u,t\right)=f_{p}\left(u\left(t\right)\right)$, can be reconstructed from fossil samples of $\bar{y}\left(t\right)$, and provided that this function is sufficiently smooth this can be done also when the fossil samples are sparse and at irregular time intervals. For the bryozoan data in Figure 1, it turns for example out that the mean trait value $\bar{y}=\log\;\mathrm{AZ}\;\text{area mean}$ as function of the environment $u=\partial^{18}O$ can be modeled by a straight line, from which follows continuous predictions $\hat{\bar{y}}$ as function of time (see Section 4 for details).

The prediction method outlined above is supported by simulations in Section 3, with the theoretical background given in Section 2. As a basis for comparisons of different prediction models, Section 2 also includes theory for the Akaike Information Criterion (AIC). The simulations are based on a reaction norm model where I use the $\partial^{18}O\left(t\right)$ measure as environmental cue, and where the phenotypic plasticity may be set to zero. This gives a connection between the simulations and the real bryozoan data case in Section 4, in the sense that the same type of large but short‐time fluctuations in the input signal is used. Since both $\partial^{18}O\left(t\right)$ and $\bar{y}\left(t\right)$ in the simulations have large variances, and since data from the fossil record often are mean values over both populations and time (Liow et al. 2024), I will in the simulations use moving average filtered versions of these variables. The simulations aim to show that mean phenotype versus environment functions can be predicted from sparse data also in cases where the population in shorter time periods have quite low mean fitness. The simulations also show that $\bar{y}\left(t\right)$ fluctuates around the optimal value $\theta_{p}\left(u,t\right)$, as discussed above, although in some cases with a minor time lag. In order to show that $\theta_{p}\left(u,t\right)$ may be different from $\theta_{i}\left(u,t\right)$, a case with a constraint on individual trait values is included. In addition to theory necessary for the simulations, as given in Section 2, the theory is extended to cover multivariate cases.

In Section 4, the prediction method is applied on recently presented data from an already introduced field study of the bryozoan species *Microporella agonistes* , based on a fossil record over 2.3 million years (Liow et al. 2024). By means of a weighted least squares (WLS) method parameters in a linear mean phenotype versus environment function are here estimated from as few as nine data points at irregular sampling times. This is done using mean values of the $\partial^{18}O\left(t\right)$ measure in the time periods for collected fossil data, but also, and with better results, using moving average mean environmental data. AIC tests show that the WLS prediction model clearly outperform the alternative unbiased random walk (URW) model proposed in Liow et al. (2024). Finally follows a summary and discussion in Section 5.

## 2 Theory

### 2.1 A Basic Univariate Reaction Norm Case

For simplicity and for use in simulations I will initially assume that the population mean fitness is determined by a univariate phenotypic mean value $\bar{y}\left(t\right)$, and that the adaptive landscape thus is two‐dimensional. I will later extend some essential results to multivariate cases, and the simulations will also include examples where other factors than the physical environment are involved.

In the simulations I will make use of an individual intercept‐slope reaction norm model with parameters $a\left(t\right)$ and $b\left(t\right)$ as given by

(1) $$y\left(t\right)=a\left(t\right)+b\left(t\right)\left(u\left(t\right)-u_{\mathrm{ref}}\right)+\varepsilon \left(t\right),$$

where, $b\left(t\right)$ and the variance $G_{\mathrm{bb}}=\mathrm{var}\left(b\left(t\right)\right)$ in some cases are set to zero. Here, $u\left(t\right)$ is the environmental cue, while $\varepsilon \left(t\right)$ is assumed to be normally distributed with mean 0 and variance $\sigma_{\varepsilon }^{2}$ among individuals in every generation. In Equation (1), $u_{\mathrm{ref}}$ is a reference environment defined as the environment at which the phenotypic variance is at a minimum (Lande 2009). For a given population this will result in the mean reaction norm

(2) $$\bar{y}\left(t\right)=\bar{a}\left(t\right)+\bar{b}\left(t\right)\left(u\left(t\right)-u_{\mathrm{ref}}\right).$$

The optimal individual phenotypic value is, in general, a function of the environment

(3) $$\theta_{i}\left(u,t\right)=f\left(u\left(t\right)-u_{\mathrm{ref}}\right),$$

in the linear case $\theta_{i}\left(u,t\right)=\alpha +\beta \left(u\left(t\right)-u_{\mathrm{ref}}\right)$, where α and β are constant parameters.

In the simulations I will assume an individual fitness function

(4a) $$W\left(t\right)=W_{\max}\;\exp\left(-\frac{{\left(y\left(t\right)-\theta_{i}\left(u,t\right)\right)}^{2}}{2\omega^{2}}\right),$$

which assuming that $y\left(t\right)$ is normally distributed gives the mean fitness in a population (Lande 2009; Estes and Arnold 2007).

(4b) $$\bar{W}\left(t\right)=W_{\max}\sqrt{\frac{\omega^{2}}{\omega^{2}+\sigma_{y}^{2}\left(t\right)}}\exp\left(-\frac{{\left(\bar{y}\left(t\right)-\theta_{i}\left(u,t\right)\right)}^{2}}{2\left(\omega^{2}+\sigma_{y}^{2}\left(t\right)\right)}\right),$$

where, $\sigma_{y}^{2}\left(t\right)$ is the variance of $y\left(t\right)$ according to Equation (1). The mean fitness will thus be maximized when $\bar{y}\left(t\right)=\theta_{i}\left(u,t\right)$. Note that this is an example with symmetrical individual fitness and trait distribution functions, such that optimal individual and mean phenotypic values are equal, that is, $\theta_{p}\left(u,t\right)=\theta_{i}\left(u,t\right)$. In Section 3.3 I will, however, also include a case where, $\theta_{p}\left(u,t\right)\neq \theta_{i}\left(u,t\right)$.

Under the assumptions of non‐overlapping generations and random mating in a large population, the evolution of the mean reaction norm parameters is governed by (Lande 1979).

(5) $$\left[\begin{array}{c} \bar{a}\left(t+1\right)\\ \bar{b}\left(t+1\right) \end{array}\right]=\left[\begin{array}{c} \bar{a}\left(t\right)\\ \bar{b}\left(t\right) \end{array}\right]+\left[\begin{array}{cc} G_{\mathrm{aa}} & G_{\mathrm{ab}}\\ G_{\mathrm{ab}} & G_{\mathrm{bb}} \end{array}\right]{\left[\begin{array}{cc} G_{\mathrm{aa}}+\sigma_{\varepsilon }^{2} & G_{\mathrm{ab}}\\ G_{\mathrm{ab}} & G_{\mathrm{bb}} \end{array}\right]}^{-1}\;\left[\begin{array}{c} \mathrm{cov}\left(w\left(t\right),a\left(t\right)+\varepsilon \left(t\right)\right)\\ \mathrm{cov}\left(w\left(t\right),b\left(t\right)\right) \end{array}\right],$$

where, $G_{\mathrm{aa}}$, $G_{\mathrm{bb}}$, and $G_{\mathrm{ab}}$ are variances and the covariance of the reaction norm parameters, and where $w\left(t\right)=W\left(t\right)/\bar{W}\left(t\right)$ is the relative fitness. In a stationary stochastic environment, the optimal reaction norm slope is ${\bar{b}}_{\mathrm{opt}}=\mathrm{cov}\left(\theta_{i}\left(u,t\right),u\left(t\right)\right)/\mathrm{var}\left(u\left(t\right)\right)$ (Lande 2009).

When the mean phenotypic trait in a population is tracking a moving optimum $\theta_{p}\left(u,t\right)$ it will be given by $\bar{y}\left(t\right)=\theta_{p}\left(u,t\right)-e\left(t\right)$, where $e\left(t\right)$ is the tracking error (see block diagram in Figure 2). This means that the parameter values $\bar{a}\left(t\right)$ and $\bar{b}\left(t\right)$ are forced to evolve such that $\bar{y}\left(t\right)\approx \theta_{p}\left(u,t\right)=\theta_{i}\left(u,t\right)=f\left(u\left(t\right)-u_{\mathrm{ref}}\right)$, that is, such that the mean fitness according to Equation (4b) is kept close to the optimum and that the tracking error is small. In feedback control terminology it is thus the mean trait tracking error $e\left(t\right)=\theta_{p}\left(u,t\right)-\bar{y}\left(t\right)$ that via mean fitness is the driving force in the adaptive process. It follows from Equation (5) that the tracking system in Figure 2 has what in feedback control terminology is called integral action (Åström and Murray 2008), which means that the tracking error in a stationary case with a fixed adaptive peak will asymptotically go to zero. As shown in simulations in Section 3 the parameters in the $\bar{y}\left(t\right)\approx \theta_{p}\left(u,t\right)=f\left(u\left(t\right)-u_{\mathrm{ref}}\right)$ function can be estimated also from rather few fossil data points $\left[u_{\text{filt}}\left(t\right),{\bar{y}}_{\text{filt}}\left(t\right)\right]$ at irregular sampling times, where ${\bar{y}}_{\text{filt}}\left(t\right)$ and $u_{\text{filt}}\left(t\right)$ are moving average filtered versions of often quite noisy signals. In the real data case in Section 4 the parameters in $\theta_{p}\left(u,t\right)$ are found from data points $\left[u_{\text{filt}}\left(t\right),\bar{y}\left(t\right)\right]$, but also from $\left[u_{\text{mean}}\left(t\right),\bar{y}\left(t\right)\right]$, where $u_{\text{mean}}\left(t\right)$ is the environmental mean over the time periods for collected fossil data. Note that even though $\bar{y}\left(t\right)\approx f\left(u\left(t\right)-u_{\mathrm{ref}}\right)$, such a model is not a reaction norm, that is, the reaction to a sudden change ∆u in $u\left(t\right)$ will still be a sudden change $∆\bar{y}=\bar{b}\left(t\right)∆u$.

The details in the tracking process in Figure 2 as used in the simulations are described by Equations (2), (4) and (5), but also if the parameters in those equations were known they could not be used for computation of predictions $\hat{\bar{y}}\left(t\right)$ based on only a few fossil samples at irregular time points. The remaining possibility for prediction of $\bar{y}\left(t\right)$ is to assume good enough tracking such that $\bar{y}\left(t\right)\approx \theta_{p}\left(u,t\right)=f\left(u\left(t\right)-u_{\mathrm{ref}}\right)$, which as shown in the simulations makes it possible to estimate the parameters in the $\theta_{p}\left(u,t\right)$ function also from few $\left[u_{\text{filt}}\left(t\right),{\bar{y}}_{\text{filt}}\left(t\right)\right]$ or $\left[u_{\text{mean}}\left(t\right),{\bar{y}}_{\text{mean}}\left(t\right)\right]$ data points. Based on those estimates and known values of $u\left(t\right)$ it is then possible to predict $\bar{y}\left(t\right)$.

### 2.2 Influence of Other Factors Than the Environment

Equations (2) and (3) assume that $u\left(t\right)$ is the only factor that affects the mean phenotypic value $\bar{y}\left(t\right)$ and the optimal mean phenotypic value $\theta_{p}\left(u,t\right)=\theta_{i}\left(u,t\right)$, respectively. When other factors affect $\bar{y}\left(t\right)$, the tracking system in Figure 2 will force the parameters $\bar{a}\left(t\right)$ and $\bar{b}\left(t\right)$ in Equation (2) to evolve such that these factors are compensated for, and the result will be seen in the mean phenotype versus environment function that can be found from data, and then most likely in the form of nonlinearities as shown in a simulation example in Section 3.3. When other factors affect $\theta_{p}\left(u,t\right)$ it is again the $\bar{a}\left(t\right)$ and $\bar{b}\left(t\right)$ parameter values that must compensate, also then most likely in the form of nonlinearities as shown in Section 3.4. A linear mean phenotype versus environment function thus indicates that no other factors than the environment affect the fitness versus environment function, although there may of course be several other factors that compensate the effects of each other. And in cases where $\theta_{i}\left(u,t\right)$ is a nonlinear function there may possibly be factors that make $\theta_{p}\left(u,t\right)$ linear.

### 2.3 Multivariate Cases

When the mean phenotypic trait in a univariate population is tracking the optimal phenotypic value, the mean phenotypic trait can, as shown above, be described by $\bar{y}\left(t\right)\approx \theta_{p}\left(u,t\right)=f\left(u\left(t\right)-u_{\mathrm{ref}}\right)$, that is, by the adaptive peak versus environment function. Here, it is again important to note that we do not need to know the details of the tracking process in Figure 2.

The univariate theory can be extended to multivariate cases, and although we in practice do not need to know the underlying fitness function, we may for a theoretical discussion use an example with two phenotypic traits and the bivariate fitness function (Johnson and Wichern 2008, Ch. 4)

(6) $$W\left(t\right)=W_{\max}\;\exp\left(-\left[y_{1}\left(t\right)-\theta_{i,1}\left(u,t\right)\;y_{2}\left(t\right)-\theta_{i,2}\left(u,t\right)\right]\Sigma^{-1}\left(t\right)\left[\begin{array}{c} y_{1}\left(t\right)-\theta_{i,1}\left(u,t\right)\\ y_{2}\left(t\right)-\theta_{i,2}\left(u,t\right) \end{array}\right]/2c\right),$$

where, $\Sigma \left(t\right)=\mathrm{cov}\left(y_{1}\left(t\right),y_{2}\left(t\right)\right)$ is the variance–covariance matrix and where c is a scaling factor controlling the “width” of the fitness function. In order to keep the mean traits in the vicinity of the adaptive peak, they must in this case continuously evolve such that ${\bar{y}}_{1}\left(t\right)\approx \theta_{p,1}\left(u,t\right)$ and ${\bar{y}}_{2}\left(t\right)\approx \theta_{p,2}\left(u,t\right)$, and we will thus find

(7a) $${\bar{y}}_{1}\left(t\right)\approx f_{1}\left(u\left(t\right)-u_{\mathrm{ref}}\right)$$

(7b) $${\bar{y}}_{2}\left(t\right)\approx f_{2}\left(u\left(t\right)-u_{\mathrm{ref}}\right),$$

where, the moving adaptive peak versus environment functions may be linear or nonlinear, and where the parameters can be estimated from rather few fossil data points at irregular sampling times. Here, the essential point is that ${\bar{y}}_{1}\left(t\right)$ and ${\bar{y}}_{2}\left(t\right)$ in this example are tracking a peak in a three‐dimensional adaptive landscape, and that this tracking process can be decomposed into two univariate tracking processes. We may thus consider the projections of the bivariate fitness function onto orthogonal axes for the two phenotypic traits, and such a projection approach may be extended to systems with higher dimensions, as discussed in Goodnight (2013). Note that we here are only interested in the $\theta_{p,1}\left(u,t\right)$ and $\theta_{p,2}\left(u,t\right)$ functions that define the position of the adaptive peak, and that we do not need to know the shape of the adaptive landscape around the peak.

### 2.4 Theory for the Akaike Information Criterion

#### 2.4.1 AIC Formulation

The AIC is given by Banks and Joyner (2017)

(8a) $$\mathrm{AIC}=2k-2\ln\left(L\left({\hat{\Theta }}_{\mathrm{MLE}}|y\right)\right),$$

where, k is the number of estimated parameters, while $L\left({\hat{\Theta }}_{\mathrm{MLE}}|y\right)$ is the likelihood of the estimated parameters using maximum likelihood estimation, given the vector y of observed data. For small sample sizes N a penalty term should be added, such that

(8b) $${\mathrm{AIC}}_{c}=\mathrm{AIC}+\frac{2k\left(k+1\right)}{N-k-1},$$

#### 2.4.2 AIC for Weighted Least Squares Estimation

Assume a statistical model for a random variable

(9) $$Y_{j}=f\left(t_{j},q\right)+w_{j}E_{j},$$

where, $w_{j}$ are known weights and where $E_{j}$ for j=1,2,…,N is i.i.d. $Ν\left(0,\sigma^{2}\right)$, while q is a parameter vector. Under this model we find (Banks and Joyner 2017)

(10) $$\mathrm{AIC}=2k+\mathrm{Nln}\left(2\pi \right)+N+2\sum_{j=1}^{N}\ln\left(w_{j}\right)+\mathrm{Nln}\left(\frac{\sum_{j=1}^{N}w_{j}^{-2}{\left(y_{j}-f\left(t_{j},{\hat{q}}_{\mathrm{WLS}}\right)\right)}^{2}}{N}\right),$$

from which ${\mathrm{AIC}}_{c}$ follows according to Equation (8b).

#### 2.4.3 AIC for General and Unbiased Random Walks

Assume a random walk process where at each time step an increment of evolutionary change is drawn at random from a distribution of evolutionary steps, and that this distribution is normal with mean value $\mu_{\text{step}}$ and variance $\sigma_{\text{step}}^{2}$. For an observed evolutionary change ΔX over T time steps the log‐likelihood function for a general random walk (GRW) process is given by Hunt (2006)

(11) $$\ln\left(\mu_{\text{step}},\sigma_{\text{step}}^{2}\right)=-\frac{1}{2}\ln\left(2\pi \right)-\frac{1}{2}\ln\left(T\sigma_{\text{step}}^{2}+\frac{V_{p_{A}}}{n_{A}}+\frac{V_{p_{D}}}{n_{D}}\right)-\frac{{\left(\Delta X-T\mu_{\text{step}}\right)}^{2}}{2\left(T\sigma_{\text{step}}^{2}+\frac{V_{p_{A}}}{n_{A}}+\frac{V_{p_{D}}}{n_{D}}\right)},$$

where, $n_{A}$ and $n_{D}$ are the number of observed ancestors and descendants, respectively, while $V_{p_{A}}$ and $V_{p_{D}}$ are the corresponding population phenotypic variances. This log‐likelihood function can be maximized by a numerical search for optimal $\mu_{\text{step}}$ and $\sigma_{\text{step}}^{2}$ values, as done in Section 4.3. When $\mu_{\text{step}}$ is fixed to zero we obtain the URW process.

## 3 Simulations

### 3.1 Linear Reaction Norm Example

A system with a linear mean reaction norm $\bar{y}\left(t\right)=\bar{a}\left(t\right)+\bar{b}\left(t\right)\left(u\left(t\right)-u_{\mathrm{ref}}\right)$ was simulated with use of the 2115 available samples of $u\left(t\right)=\partial^{18}O\left(t\right)$, and with population size 400. These samples go 5.3 million years back in time with various sampling intervals (Lisiecki and Raymo 2005), but here I just assume 2115 nonoverlapping generations (however, see Section 5 for a discussion on relevance for fossil data). At each new generation random individual samples around $\bar{a}\left(t\right)$, $\bar{\varepsilon }\left(t\right)=0$ and $\bar{b}\left(t\right)$ were drawn from normal populations with the given variances. The fitness function in Equation (4a) had parameter values $W_{\max}=1$ and $\omega^{2}=50$. The optimal mean phenotypic value was in a first linear case chosen as $\theta_{p}\left(u,t\right)=\theta_{i}\left(u,t\right)=u\left(t\right)-2.9$, where $\theta_{p}\left(u,t\right)$ thus and for clarity of presentation is assumed to be a deterministic function, and where 2.9 is the mean value of $\partial^{18}O\left(t\right)$ around t=0 (however, see results with added white noise below).

As it is not realistic that $u\left(t\right)$ and $\theta_{p}\left(u,t\right)$ are purely deterministic signals, correlated white noise with variances $\sigma_{u}^{2}=0.05$ and $\sigma_{\theta }^{2}=0.2$, respectively, were added to $\theta_{p}\left(u,t\right)$ and $u\left(t\right)$. The covariance was $\sigma_{\theta ,u}=0.025$, see Lande (2009) for a possible explanation.

**FIGURE 3:**
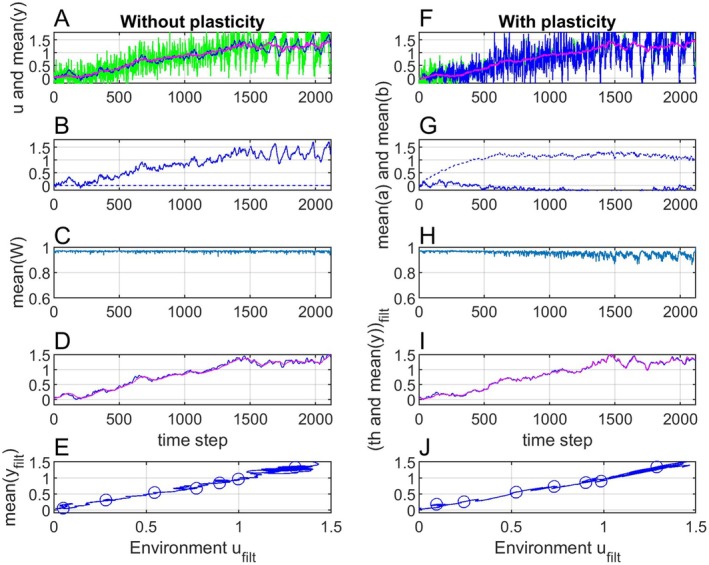
Simulation results with the linear $\theta_{p}\left(u,t\right)=\theta_{i}\left(u,t\right)=u\left(t\right)-2.9$ function, without plasticity (panels A–E) and with plasticity (panels F–J). Panels A and F show $u\left(t\right)$ (green lines) and $\bar{y}\left(t\right)$ (blue lines), while the red line is $u_{\text{filt}}\left(t\right)$. Panels B and G show $\bar{a}\left(t\right)$ (solid lines) and $\bar{b}\left(t\right)$ (dashed lines), while panels C and H show mean fitness $\bar{W}\left(t\right)$. Panels (D and I) show $\theta_{p,\text{filt}}\left(u,t\right)$ (blue lines) and ${\bar{y}}_{\text{filt}}\left(t\right)$ (red lines). Panels (E and J) show that ${\bar{y}}_{\text{filt}}\left(t\right)$ as function of $u_{\text{filt}}\left(t\right)$ in both cases is the same straight line, with values for samples 200, 400, 600, 800, 1000, 1200, and 1400 shown by circles.

In a first case without phenotypic plasticity, the reaction norm parameter variances and covariance were chosen as $G_{\mathrm{aa}}=2$, $\sigma_{\varepsilon }^{2}=0.5\;G_{\mathrm{aa}}=1$, $G_{\mathrm{bb}}=0$ and $G_{\mathrm{ab}}=0$, while the initial parameter values were $\bar{a}\left(0\right)=\bar{b}\left(0\right)=0$. Since $\bar{y}\left(t\right)$, $\theta_{p}\left(u,t\right)$ and $u\left(t\right)=\partial^{18}O\left(t\right)$ have large variances, I used moving average filtered signals ${\bar{y}}_{\text{filt}}\left(t\right)$, $\theta_{p,\text{filt}}\left(u,t\right)$ and $u_{\text{filt}}\left(t\right)$ in some of the plots. These were obtained by moving window filters with window size 100, in MATLAB notation ${\bar{y}}_{\text{filt}}\left(t\right)=\text{movmean}\left(\bar{y}\left(t\right),100\right)$, $\theta_{p,\text{filt}}\left(u,t\right)=\text{movmean}\left(\theta_{p}\left(u,t\right),100\right)$ and $u_{\text{filt}}\left(t\right)=\text{movmean}\left(u\left(t\right),100\right)$. Simulation results in the linear case without plasticity are shown in Figure 3A–E. Note that $\bar{y}\left(0\right)=\bar{a}\left(t\right)$ follows the main trend in $u\left(t\right)$, and that the fluctuations are somewhat delayed compared to the fluctuations in $u\left(t\right)$ (panel A).

In a second case with phenotypic plasticity, the reaction norm parameter variances and covariance were chosen as $G_{\mathrm{aa}}=2$, $\sigma_{\varepsilon }^{2}=1$, $G_{\mathrm{bb}}=2$ and $G_{\mathrm{ab}}=0$, while the initial parameter values were $\bar{a}\left(0\right)=0$ and $\bar{b}\left(0\right)=0.8$. Simulation results in the linear case with plasticity are shown in Figure 3F–J. As follows from the reaction norm Equations (1) and (2), the variance $\sigma_{y}^{2}\left(t\right)$ will increase with $u\left(t\right)-u_{\mathrm{ref}}$, and as a result the mean fitness will be reduced (panel H).

In both cases (without and with plasticity) ${\bar{y}}_{\text{filt}}\left(t\right)$ tracks $\theta_{p,\text{filt}}\left(u,t\right)=\theta_{i,\text{filt}}\left(u,t\right)$ closely (panels D and I), such that the mean fitness is kept at a high level (panels C and H). This results in linear functions ${\bar{y}}_{\text{filt}}\left(t\right)=f\left(u_{\text{filt}}\left(t\right)\right)$ (panels E and J), with seven discrete values marked by circles. Using $u_{\mathrm{ref}}=u_{\text{filt}}\left(0\right)=2.9$, those discrete values could be used for determination of the parameters $\hat{\alpha }$ and $\hat{\beta }$ of this line by an ordinary or WLS method, and thus to find predictions ${\hat{\bar{y}}}_{\text{filt}}\left(t\right)=\hat{\alpha }+\hat{\beta }\left(u_{\text{filt}}\left(t\right)-u_{\mathrm{ref}}\right)$, see examples in Section 4.

### 3.2 Nonlinear Reaction Norm Example

**FIGURE 4:**
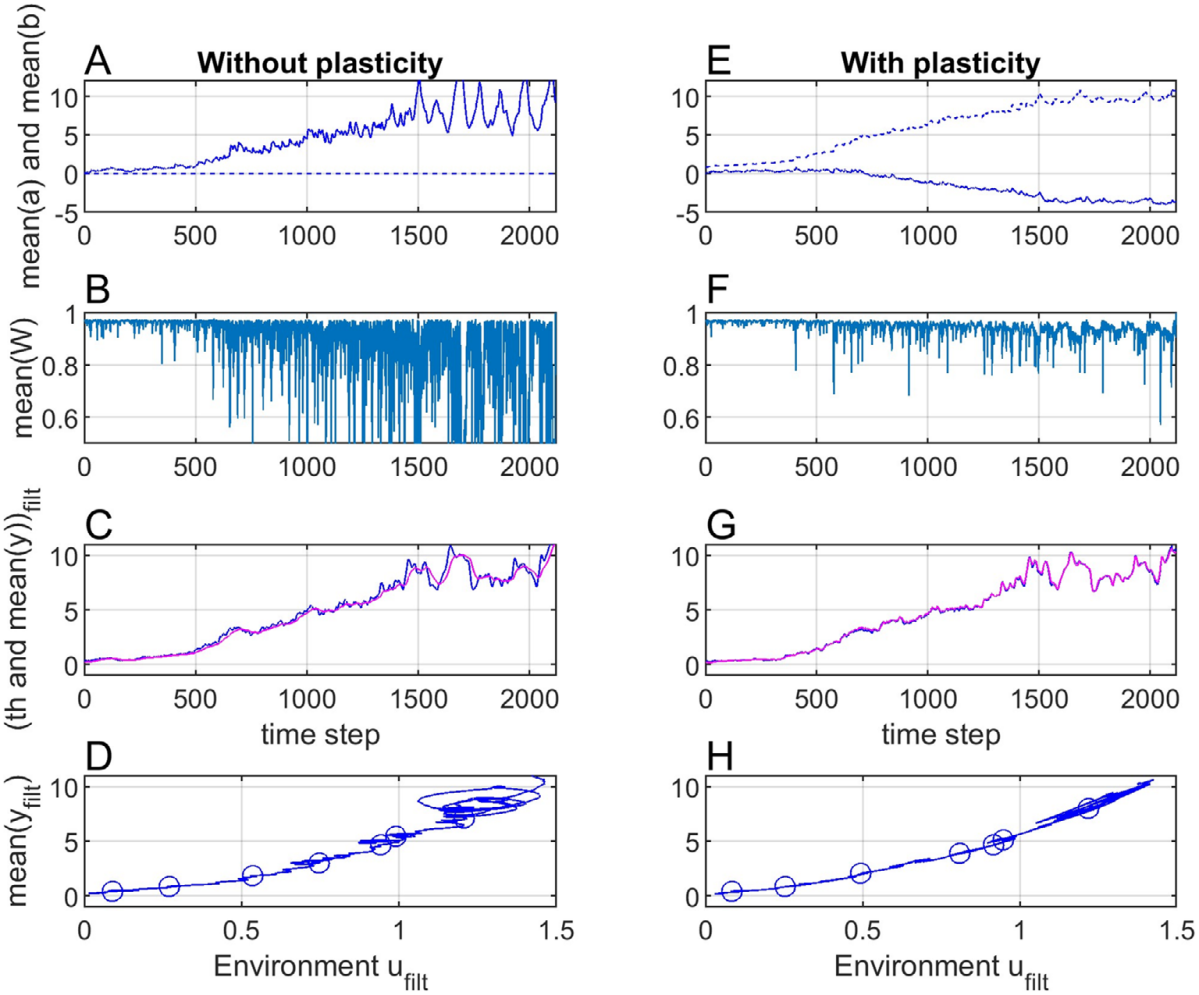
Simulation results as in Figure 3, but with $\theta_{p}\left(u,t\right)=\theta_{i}\left(u,t\right)=\left(u\left(t\right)-2.9\right)+4{\left(u\left(t\right)-2.9\right)}^{2}$. Panels A–D correspond to panels B–E in Figure 3, while panels E–H correspond to panels G–J. The discrete values in panels D and H could here be used to determine the nonlinear function ${\hat{\bar{y}}}_{\text{filt}}\left(t\right)=f\left(u_{\text{filt}}\left(t\right)\right)$ by means of any suitable fitting method.

Figure 4 shows results as in Figure 3, but with the nonlinear adaptive peak versus environment function $\theta_{p}\left(u,t\right)=\theta_{i}\left(u,t\right)=\left(u\left(t\right)-2.9\right)+4{\left(u\left(t\right)-2.9\right)}^{2}$. Note that the mean fitness now is reduced (panels B and F), especially in the case without plasticity. The signal ${\bar{y}}_{\text{filt}}\left(t\right)$ is also in this case tracking $\theta_{\text{filt}}\left(u,t\right)$ well, although with a noticeable time lag (panels C and G). Here, ${\bar{y}}_{\text{filt}}\left(t\right)$ as function of $u_{\text{filt}}\left(t\right)$ is the same second order function in the different cases (panels D and H).

### 3.3 Example With Phenotypic Constraint

**FIGURE 5:**
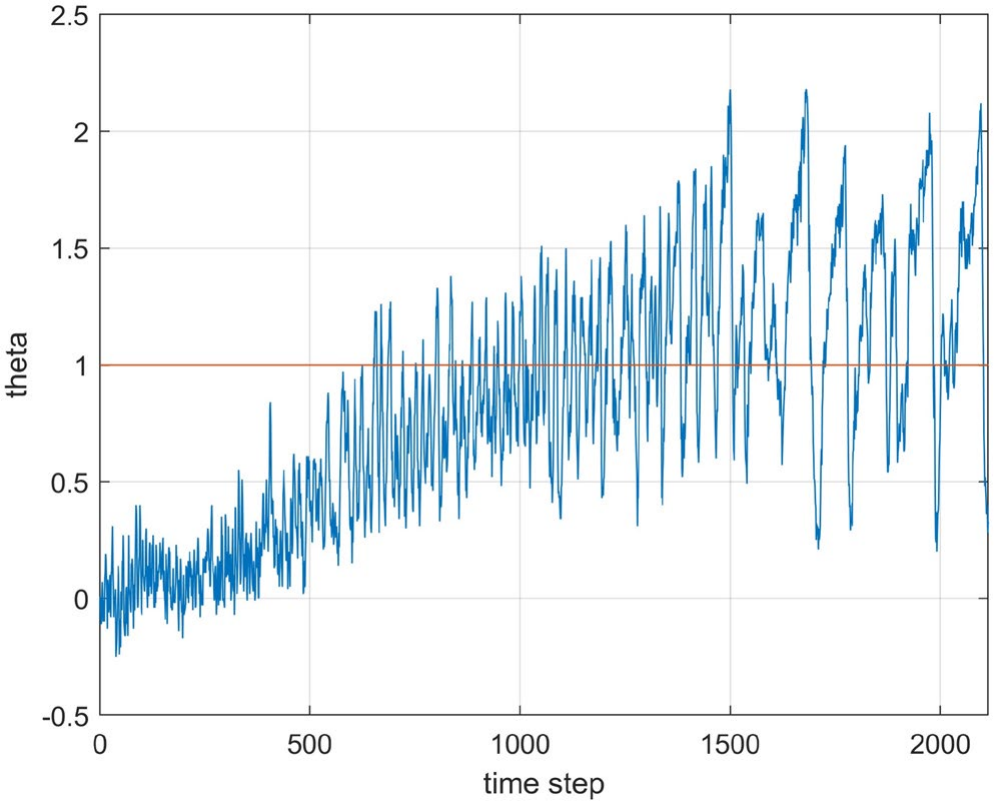
The linear function $\theta_{p}\left(u,t\right)=u\left(t\right)-2.9$ as used for the results in Figure 3 (blue line) and an upper constraint for the individual trait values (red line).

**FIGURE 6:**
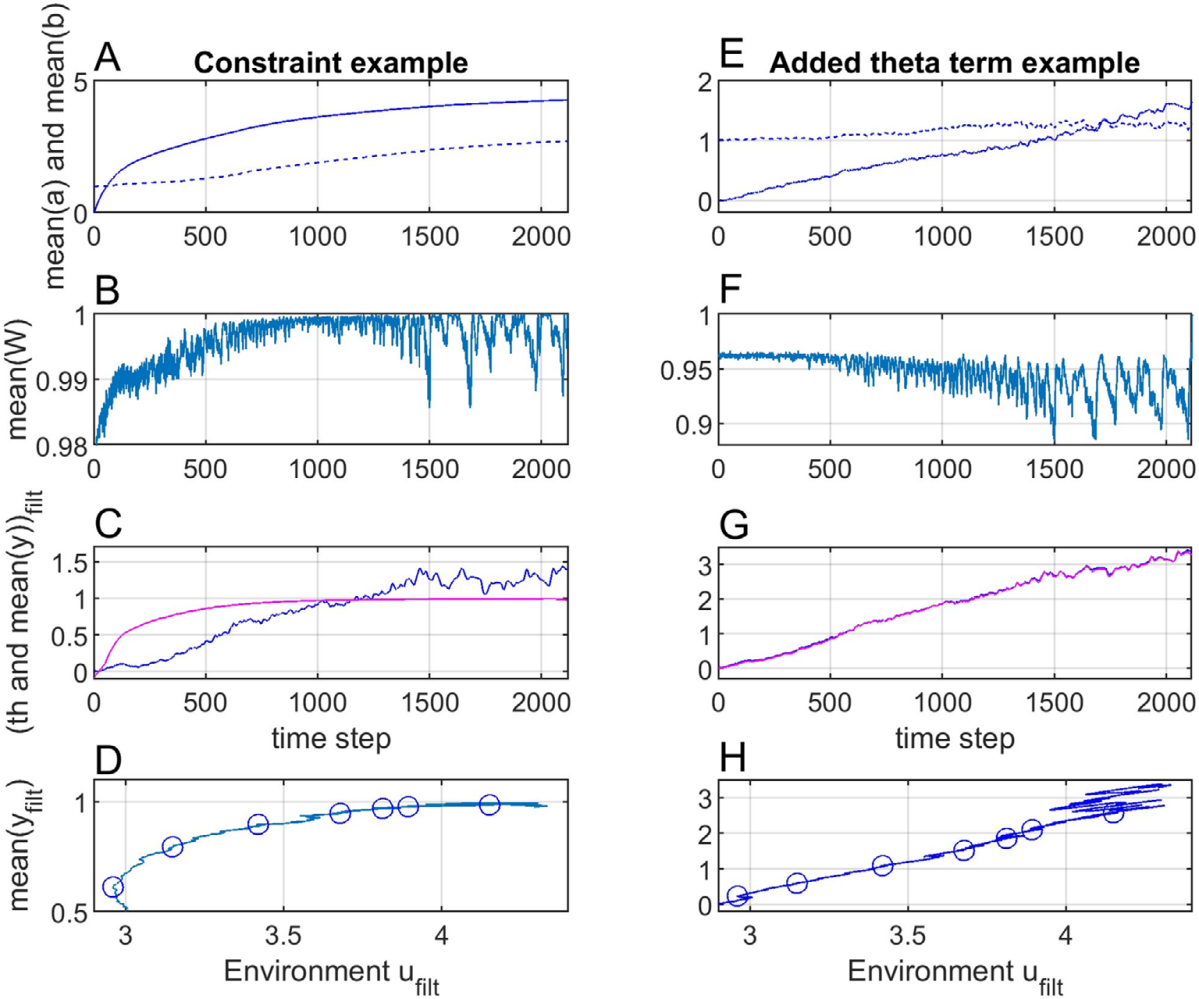
Panels (A–D) show simulation results as with plasticity in Figure 3, but with a constraint on the individual phenotypic trait values as shown in Figure 5. Panels (E–H) show simulation results as with plasticity in Figure 3, but with $\theta_{p}\left(u,t\right)=\theta_{i}\left(u,t\right)=u\left(t\right)-2.9+2t/2115$.

With a constraint on the individual phenotypic values the distribution will no longer be normal, and the adaptive peak $\theta_{p}\left(u,t\right)$ will therefore deviate from the individual fitness peak such that Equation (4b) no longer is correct. The mean trait $\bar{y}\left(t\right)$ will, however, still track the adaptive peak that maximizes mean fitness. When there for example is an upper limit for the individual phenotypic trait values as shown in Figure 5, the $\bar{a}\left(t\right)$ and $\bar{b}\left(t\right)$ parameters will evolve such that $\bar{y}\left(t\right)$ deviates from $\theta_{p}\left(u,t\right)$ with results as shown in Figure 6A–D. The effect of the constraint is clearly seen in panel C in Figure 6, in that ${\bar{y}}_{\text{filt}}\left(t\right)$ does not follow $\theta_{i,\text{filt}}\left(u,t\right)$, but instead follows $\theta_{p,\text{filt}}\left(u,t\right)$ (not shown) such that the mean fitness as shown in panel B is maximized. The effect is also seen by a comparison of panels A in Figure 6G and in Figure 3, in that $\bar{a}\left(t\right)$ evolves into a large value, such that $\bar{y}\left(t\right)$ without constraints on $y\left(t\right)$ would become larger than $\theta_{p}\left(u,t\right)$. Note that the mean fitness is increased compared to the results in Figure 3H, because the variance $\sigma_{y}^{2}\left(t\right)$ is reduced. Also note that the mean phenotype versus environment functions in panel D now is nonlinear in a way that with knowledge of the biological system possibly could be interpreted in a meaningful way. Note that individual trait values will have a larger variance than the mean trait value, such that the constraint in Figure 5 has an effect before $\bar{y}\left(t\right)\approx \theta_{p}\left(u,t\right)$ reaches the upper limit.

### 3.4 Example With Additional Term in $\theta \left(u,t\right)$

The optimal value $\theta_{p}\left(u,t\right)$ that maximizes mean fitness may not be a function of only the environment, and as an example Figure 6E–H, shows results as in Figure 3F–J, but with $\theta_{p}\left(u,t\right)=\theta_{i}\left(u,t\right)=u\left(t\right)-2.9+2t/2115$. Note that panel H now shows a nonlinear function.

### 3.5 Example Without Plasticity, but With Changed Parameter Values

**FIGURE 7:**
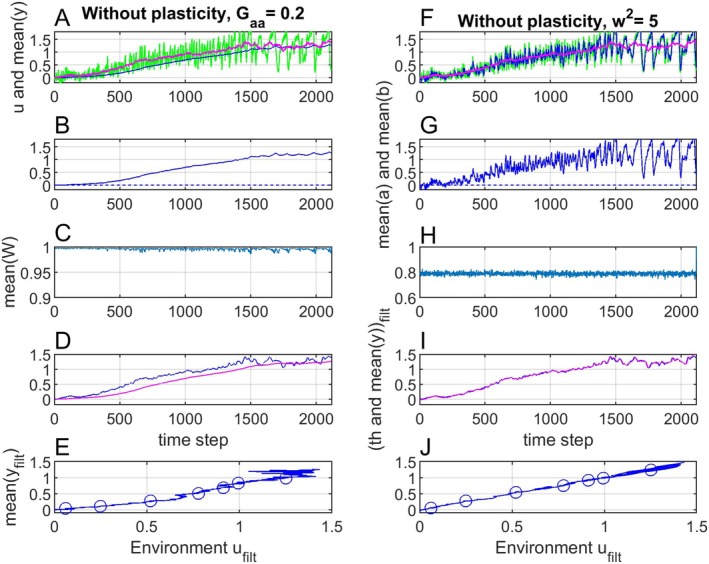
Panels (A–E) show simulation results as in Figure 3 with plasticity, but with $G_{\mathrm{aa}}$ and $\sigma_{\varepsilon }^{2}$ reduced by a factor 10. Panels (F–J) show simulation results as in Figure 3 with plasticity, but with the width of the fitness function reduced from $\omega^{2}=50$ to $\omega^{2}=5$.

Simulations as in Figure 3A–E, were repeated but with changed parameter values. First, $G_{\mathrm{aa}}$ and $\sigma_{\varepsilon }^{2}$ were reduced from 2 and 1 to 0.2 and 0.1, with results as in Figure 7A–E. As seen in panels A and F the mean phenotypic value $\bar{y}\left(t\right)$ cannot now follow the adaptive peak $\theta_{p}\left(u,t\right)$, and as a result the mean phenotype versus environment function in panel E becomes non‐linear. However, owing to the broad fitness function with $\omega^{2}=50$, the mean fitness is still close to one.

Second, with $G_{\mathrm{aa}}=2$ and $\sigma_{\varepsilon }^{2}=1$ as in Figure 3, the width of the fitness function was reduced by reducing $\omega^{2}$ from 50 to 5, with results as in Figure 7F–J. The mean phenotypic value still follows the adaptive peak but owing to the narrow fitness function the mean fitness is clearly reduced (panel H).

## 4 Real Data Case

### 4.1 Background

Liow et al. (2024) recently presented results from a field study of the bryozoan species *M. agonistes* , based on a fossil record over 2.3 million years (see Figure 1 for some results). The fossils were collected from colonies in New Zealand cliffs, and it is especially interesting that the same species still live in the waters near those cliffs. In a project running over several years, Liow et al. (2017) collected 985 fossil colonies with a large total number of individuals and developed an artificial intelligence tool for data retrieval by use of pictures from a scanning electron microscope. This image analysis system can rapidly assess three well defined phenotypic traits; autozooid (AZ) area, ovicell (OV) area and autozooid shape, and as seen from time series in figure S2 in Liow et al. (2024) the two AZ and OV area mean trait values increase with time except for a drop at present time owing to an extreme $\partial^{18}O\left(t\right)$ value.

Since the *log AZ area mean* and *log OV area mean* data in figure S2 are linearly dependent, I will here focus on the former. The last two data points in this time series are found from bryozoans from recent times with extreme $\partial^{18}O\left(t\right)$ values (figure S9 in Liow et al. 2024). As done in Figure 6 in Liow et al. (2024) these data points are therefore excluded from the analysis (see Section 5 for a discussion). The fossil data used in the analysis are thus collected from nine periods back in time according to Table 1, which for the purpose of comparison with moving average mean data also includes the time window size for 100 samples. The reason for use of the natural logarithm of the AZ area is that it gives normally distributed data (Di Martino and Liow 2021).

**TABLE 1:** Fossil collection data, using present time as zero point.

Sample number	Mean sample time point in million years	Window size in million years	Window size in million years for 100 samples	Number of colonies
1	−2.1895	0.203	0.25	102
2	−1.9660	0.102	0.25	97
3	−1.8505	0.049	0.25	14
4	−0.9265	0.019	0.2	94
5	−0.6485	0.055	0.2	202
6	−0.5480	0.030	0.2	135
7	−0.5055	0.055	0.1	182
8	−0.4510	0.054	0.1	33
9	−0.3990	0.050	0.1	17

### 4.2 Weighted Least Squares Estimation

**FIGURE 8:**
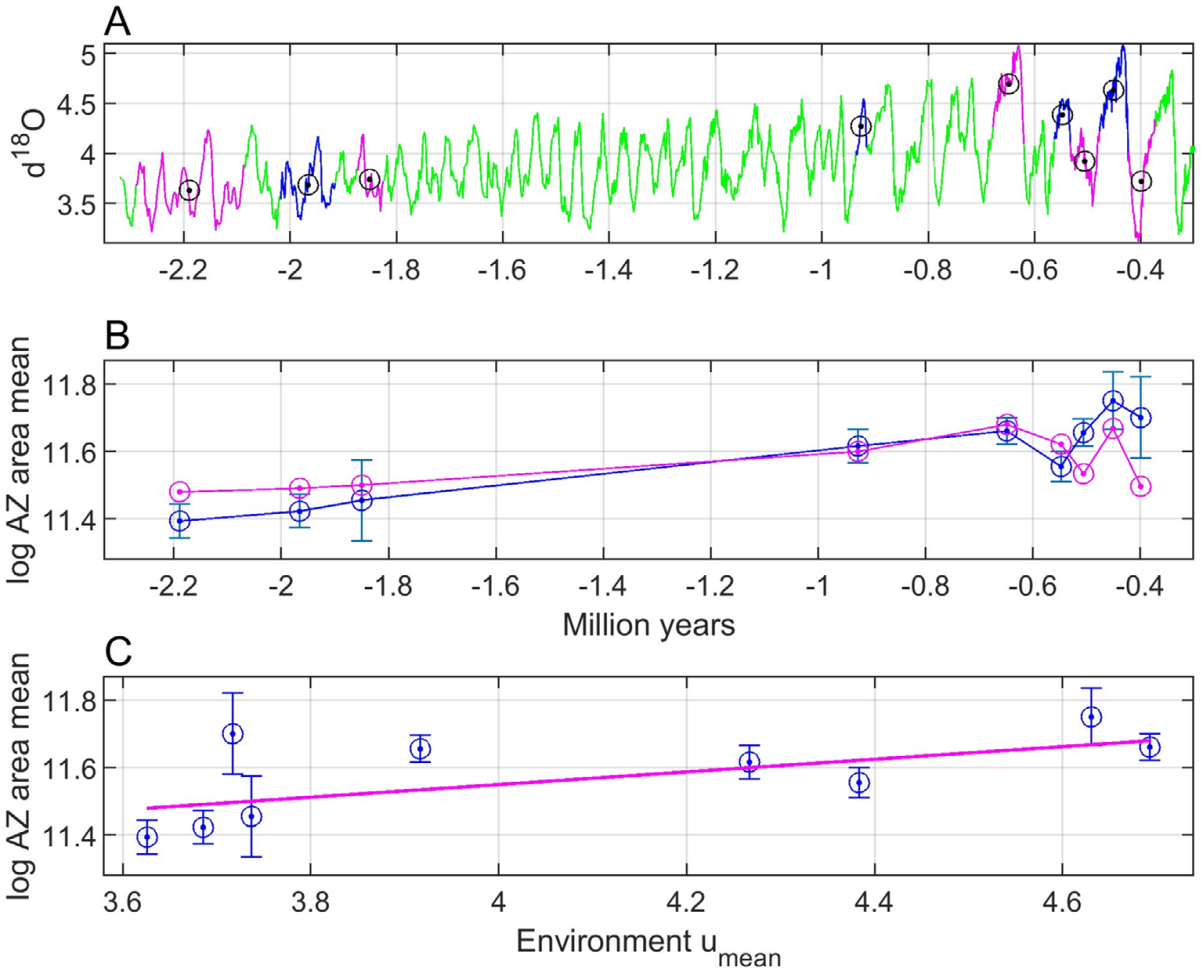
Panel (A) shows raw $\partial^{18}O\left(t\right)$ data (green line) with values in fossil collection time windows marked by red and blue lines, and with mean values marked by circles with dots. Panel (B) shows *log AZ area mean* with error bars as found from figure S2 in Liow et al. (2024) (blue circles with dots and blue line), as well as predictions (red circles with dots and red line). Panel (C) shows prediction line (red) computed by the weighted least squares method. See prediction results in Table 2.

Since the *log AZ area mean* values are given as mean values from samples within time windows as given in Table 1, it is natural to use mean values $u_{\text{mean}}\left(t\right)$ of $\partial^{18}O$ in the same windows. Figure 8 shows $u\left(t\right)=\partial^{18}O\left(t\right)$ and $y\left(t\right)=\log\;\mathrm{AZ}\;\text{area mean}\;\left(t\right),$ as well as predictions $\hat{\bar{y}}\left(t\right)$ based on WLS fitting of a straight line $y=\alpha +\beta \left(u-3.6\right)$ in a plot over $\bar{y}\left(t\right)$ as function of $u_{\text{mean}}\left(t\right)$. Here, the theoretical foundation is the statistical model in Equation (9), with each weight chosen as the reciprocal of the variance of the corresponding measurement. Note the rather large deviations from the straight line in panel C, but also that there are no indications of a non‐linear function. The slope of the prediction line is 0.19, as in Figure 6 in Liow et al. (2024), and the mean squares prediction error $\mathrm{MSE}={\left(\bar{y}\left(t\right)-\hat{\bar{y}}\left(t\right)\right)}^{2}/\left(\text{number of samples}\right)$ is MSE=0.0089 (Table 2).

**TABLE 2:** Results with different prediction models.

	Model with use of $u_{\text{mean}}$ as in Figure 8	Model with use of $u_{\text{filt}}$ as in Figure 9, window size 100	Model with use of $u_{\text{filt}}$ as in Figure 9, window size 800
β	0.19	0.52	0.51
MSE	0.0091	0.0016	0.0022
$b_{\mathrm{CV}}$	—	0.52	—
${\mathrm{MSE}}_{\mathrm{CV}}$	—	0.0020	—

**FIGURE 9:**
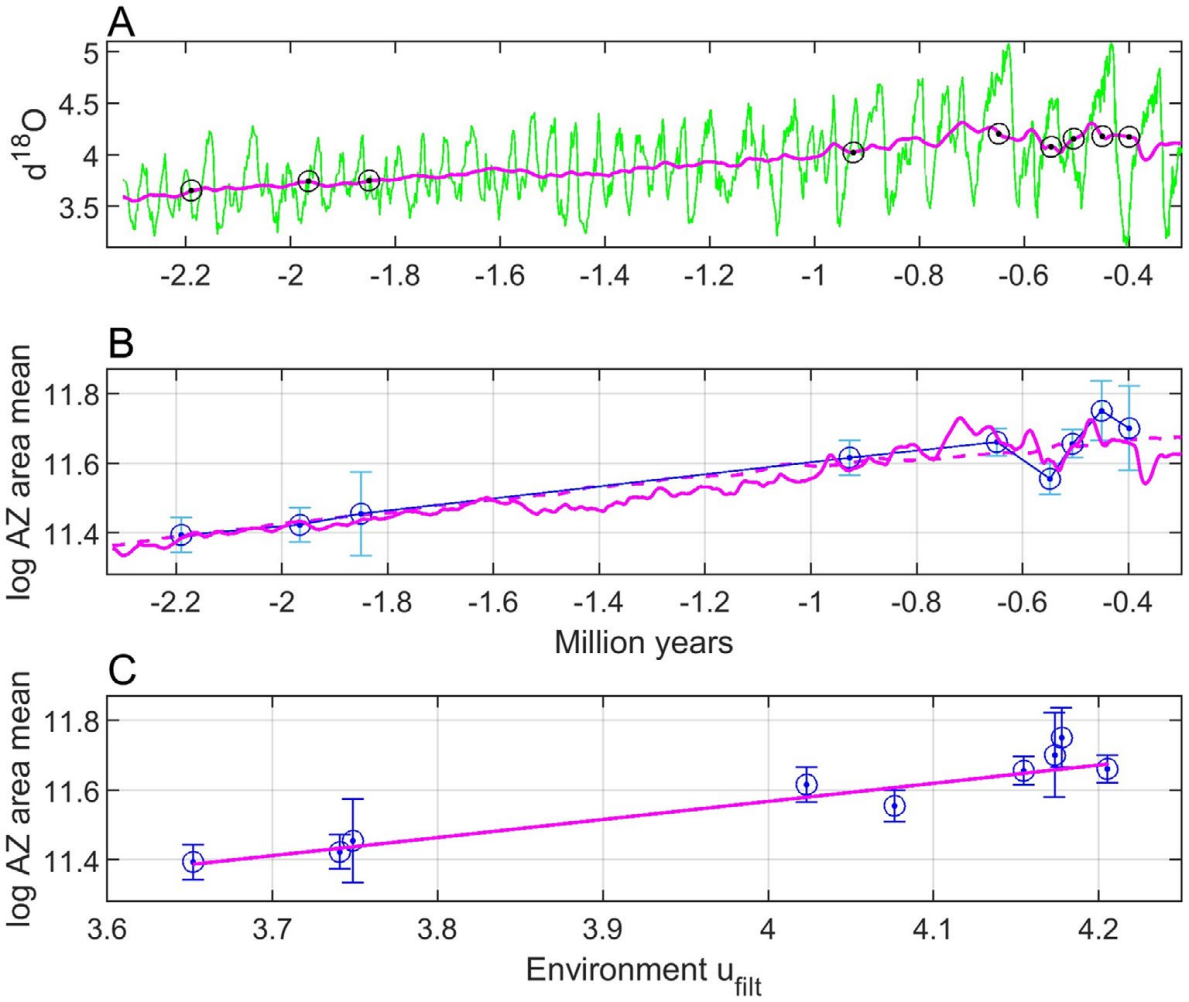
Results as in Figure 8, but based on centered moving average filtering of $\partial^{18}O\left(t\right)$ with window size 100 samples, and with continuous predictions of *log AZ mean area* values (red line in panel B). Predictions with window size 800 are added (dashed red line in panel B). See prediction results in Table 2.

Figure 9 shows the corresponding results based on centered moving average filtering of $\partial^{18}O\left(t\right)$. The optimal moving window size was 100 samples, resulting in prediction line slope 0.52 and prediction error ${\mathrm{MSE}}_{\min}=0.0016$. Note that the deviations from the straight line in panel C are clearly smaller than in Figure 8. Also note that it from the way the $\partial^{18}O\left(t\right)$ data are organized in Lisiecki and Raymo (2005) follows that the number of 100 samples corresponds to different time windows as shown in Table 1.

**FIGURE 10:**
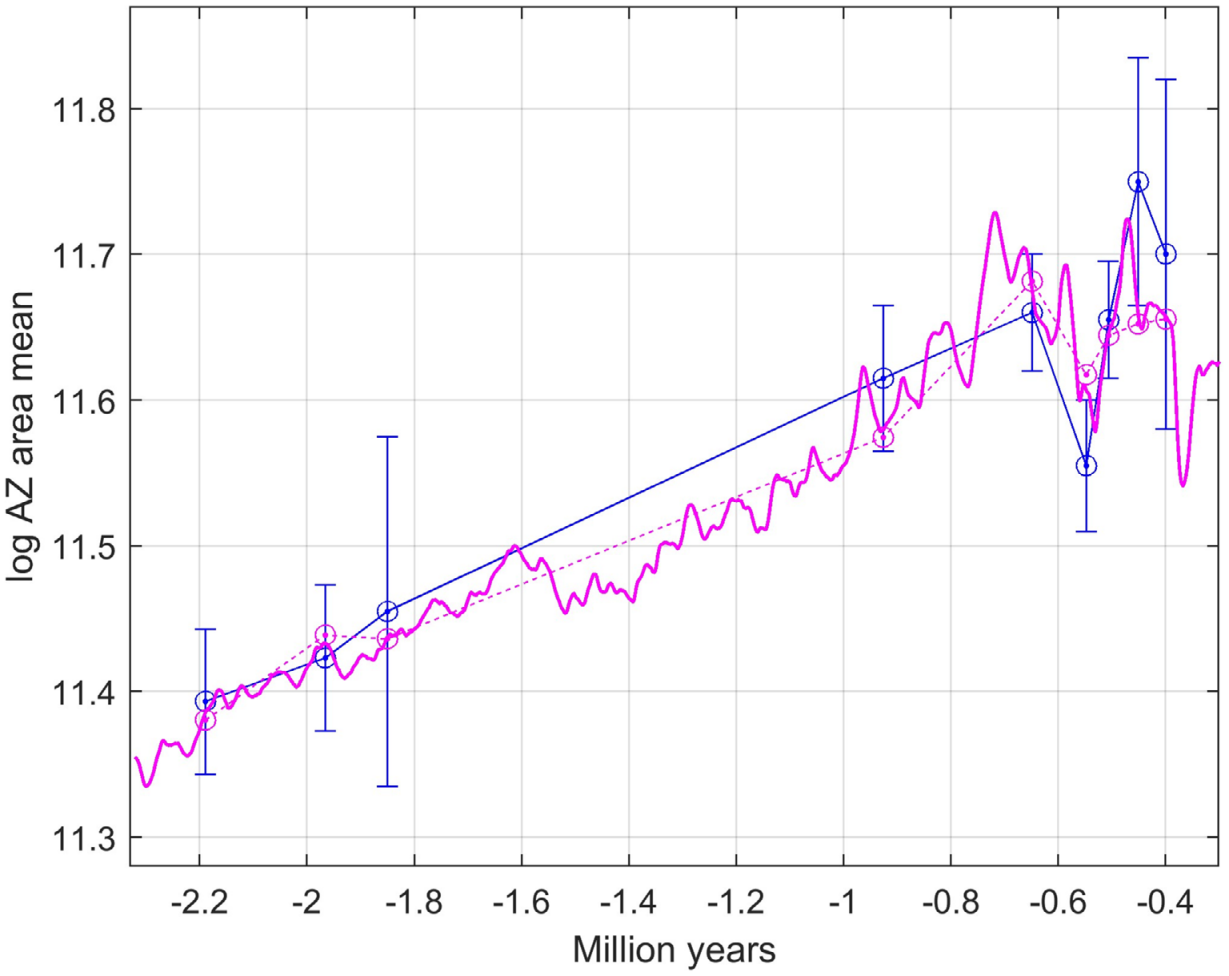
Results as in Figure 9B, and for leave‐one‐out cross validation. Solid red line shows prediction results, while dashed red line and red circled with dots show validation results for the left‐out samples. See prediction and validation results in Table 2.

The prediction results for window size 100 in Figure 9 were validated by leave‐one‐out cross validation, with results as in Figure 10 and Table 2.

**FIGURE 11:**
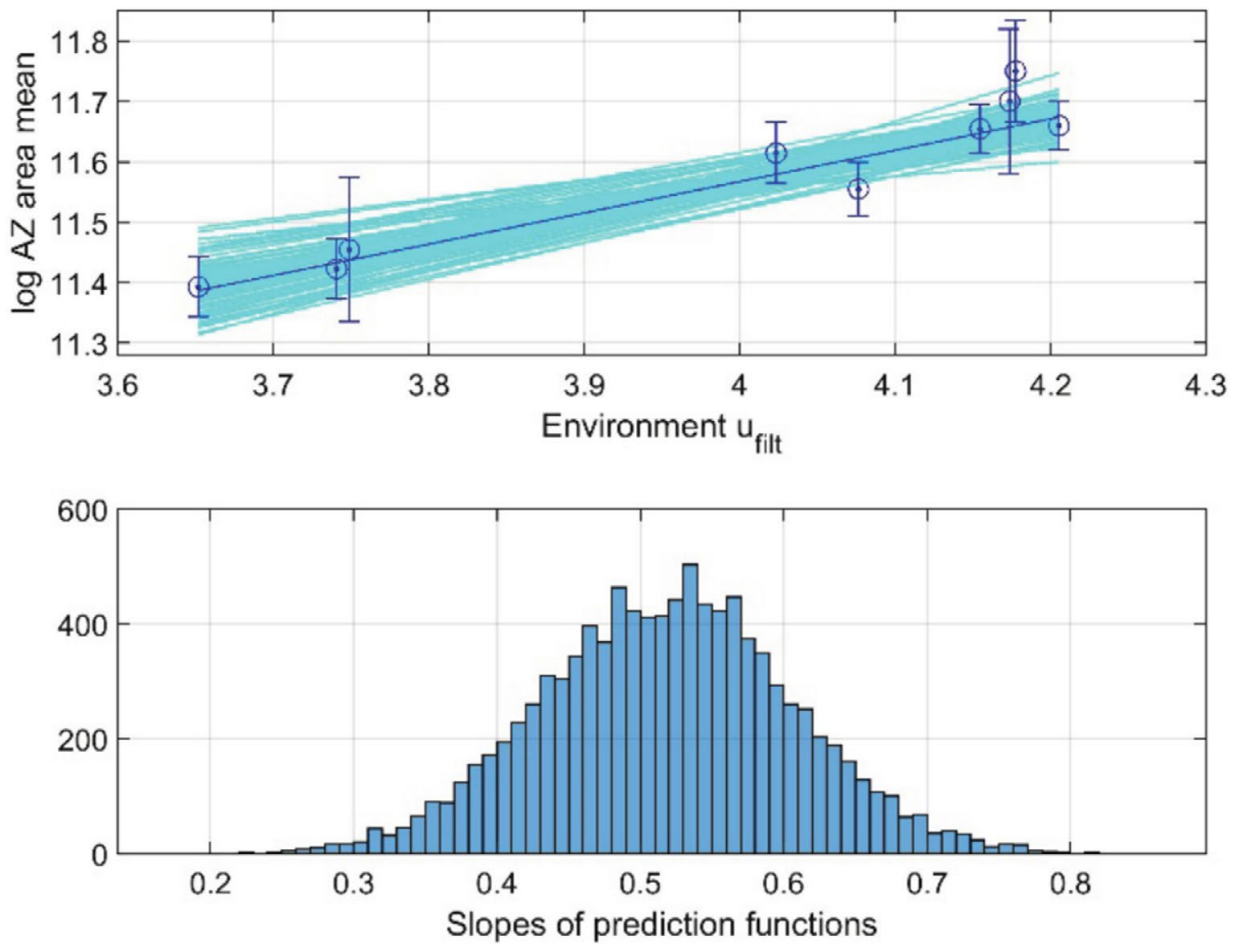
Upper panel shows linear prediction lines for *log AZ area mean* from 100 simulations with measurement values drawn from normal distributions of $\bar{y}\left(t\right)$ with the given standard errors (cyan lines), and for the mean prediction line in Figure 9C (blue line with error bars). Lower panel shows histogram over slopes of fitted prediction lines as in upper panel, but for 10,000 simulations, indicating that the prediction line slope is β=0.52±0.10.

As shown in Figures 1, 8, 9, 10, the measurements of *log AZ area mean* have various standard errors. Repeated simulations with the model as used for Figure 10, and with discrete values drawn from normal distributions with standard deviations equal to the standard errors, gave results as in Figure 11. As can be seen in the upper panel there is ample space for nonlinear prediction functions, although the plot in Figure 9C, does not indicate a nonlinear function. From the histogram in the lower panel follows that the prediction line slope value β=0.52 has a standard error of approximately SE=0.10.

### 4.3 Comparison With Random Walk Processes

The WLS prediction results following from Figure 9 should be compared with results from an URW model as proposed in figure S2 in Liow et al. (2024), but also with results from a general random walk (GRW) model. The AIC results for the alternative models according to Equations ((8a), (8b), (9), (10), (11)) are summarized in Table 3, which also includes AIC values for the results following from Figure 8. Note that we for the WLS results must use k=3, since we in addition to the straight line parameters α and β implicitly estimate the variance $\sigma^{2}$ associated with Equation (9). Also note that we in the GRW and URW models have N=8 evolutionary transitions, for which the ancestor–descendant trait differences are used jointly by summing the log‐likelihoods over all observed transitions. I thus assume homogeneous dynamics and find optimal values for $\mu_{\text{step}}$ and $\sigma_{\text{step}}^{2}$ in Equation (11) that maximize this sum of $\ln\left(\mu_{\text{step}},\sigma_{\text{step}}^{2}\right)$ for all transitions (Hunt 2006). For the GRW model we thus have k=2 estimated parameters, while k=1 for the URW model. The optimal GRW and URW parameters are found by use of the MATLAB function *fmincon*, with initial parameter values sat to zero. It is unclear why the numerical search for the optimal GWR model does not result in the URW model (since it has lower AIC values), and use of other initial parameter values makes no difference.

**TABLE 3:** AIC results for alternative models.

Model	Window size	k	N	${\hat{\mu }}_{\text{step}}$	${\hat{\sigma }}_{\text{step}}^{2}$	AIC	${\mathrm{AIC}}_{c}$
${\mathrm{WLS}}_{\mathrm{Fig}.\;9}$	100	3	9	—	—	−27.9	−23.0
${\mathrm{WLS}}_{\mathrm{Fig}.\;9}$	800	3	9	—	—	−22.4	−17.6
${\mathrm{WLS}}_{\mathrm{Fig}.\;8}$	—	3	9	—	—	−11.5	−6.7
GRW	—	2	8	$175\times 10^{-6}$	$67\times 10^{-6}$	−10.9	−8.5
URW	—	1	8	0	$74\times \;10^{-6}$	−12.1	−11.5

The essential result shown in Table 3 is that the WLS model found by use of moving average filtering of the $\partial^{18}O$ data (Figure 9) clearly is the best model, while the WLS model found by use of mean $\partial^{18}O$ values for the different time windows as given in Table 1 (Figure 8) are somewhat poorer than the random walk models. It is thus the moving average filtering of the $\partial^{18}O$ signal that makes the WLS model clearly better than the random walk models.

It is interesting to note that the URW model as found in Liow et al. (2024), figure S2, is the best choice given the mean values $u_{\text{mean}}\left(t\right)$ of $\partial^{18}O$ in the different formations (time windows as shown in Table 1), and as assumed in Figure 6 in Liow et al. (2024). This is, however, also surprising, since the WLS results in Figure 9 clearly show that directional selection is at work, such that the GRW model logically should be the best. An explanation may be that the positive GRW value of ${\hat{\mu }}_{\text{step}}$ cannot explain the negative mean trait changes in transitions five and eight. As shown in Table 3 the difference between the GRW and URW models are mainly but not only caused by differences in the penalty term in ${\mathrm{AIC}}_{c}$.

## 5 Summary and Discussion

### 5.1 Main Points

In this paper I make use of two landscape metaphors. First, an individual fitness landscape where the peak of the individual fitness as a function of individual phenotypic values moves with changes in the environment. Second, an adaptive landscape where the adaptive peak (the peak of population mean fitness) as a function of mean phenotypic values moves with changes in the environment. The adaptive peak will essentially follow the individual fitness peak, but with deviations caused by non‐symmetrical fitness and trait distribution functions and possibly also owing to other causes.

I also make some other fundamental assumptions. First, species that have persisted for millions of years in a fluctuating environment have, by means of various selection mechanisms, been tracking a limited movement of an optimum in the adaptive landscape, and they have done so well enough to survive and reproduce. This assumption is supported by results in Estes and Arnold (2007) and other references in the introduction. Second, the location of the individual fitness peak is a simple continuous function of a dominating and well‐known environmental variable, for example, the temperature, such that the movement of the peak in the adaptive landscape is continuous without sudden jumps. Third, the dominating environmental variable fluctuates in a way that makes it unlikely that the mean phenotypic value gets stuck in local minima or valleys in the adaptive landscape.

Under the assumptions given, two main conclusions can be drawn from the results in this article: First, adaptive peak versus environment functions, and thus approximate mean phenotype versus environment functions can be found from sparse and short fossil data, provided that they are sufficiently spread over the time periods of interest. As demonstrated in the real data example in Section 4, the environmental data used for prediction of mean phenotypic values may be means from time periods for fossil collection or a moving average mean. Second, prediction of mean phenotypic values does not require detailed knowledge of the tracking process in Figure 2, that is, neither parameter values in an underlying reaction norm or adaptive walk model nor the fitness functions need to be known. Also note that it is not necessary to find explicit mathematical adaptive peak versus environment functions; predictions may instead be found by use of spline interpolation and similar methods.

Even if the position of the individual fitness peak is a linear or other simple function of the environment, the adaptive peak versus environment function may be less simple. This is so because non‐symmetrical fitness functions and/or trait distributions may drive the adaptive peak away from the individual fitness peak. A closer look at the tracking process might thus be necessary for interpretations of nonlinear mean phenotype versus environment results. A deeper understanding of the adaptive landscape may also be needed in cases with slow adaptation, where the mean phenotypic value can track the adaptive peak only with a large time lag.

### 5.2 Discussion of Simulation Results

The main purpose of the simulations is to show that the mean phenotypic values over time as functions of mean environment over time (panels E and J in Figure 3D,H and in Figure 4) are determined by the adaptive peak versus environment function, and thus independent of the details of the tracking process in Figure 2. The variances of the reaction norm parameters are chosen such that there are clear differences in the evolutionary responses between cases without and with plasticity. If these variances are increased by a factor 10 (or a factor 100) these differences very much disappear in that $\bar{y}\left(t\right)$ in both cases appear to follow the adaptive peak $\theta_{p}\left(t\right)=f_{p}\left(u\left(t\right)\right)$ instantaneously, while the mean phenotypic values over time as functions of mean environment over time are essentially unchanged.

Figures 3 and 4 show that although a population without plasticity may not be able to track rapid changes in the environment it may track a moving average quite well, and that the resulting mean phenotype prediction functions, linear or non‐linear, will be the same as for a population with plasticity. Note that the mean plasticity slope with a linear adaptive peak versus environment function approaches an optimal value (Figure 3F), such that the linear mean phenotype versus environment function seen in panel J can be interpreted as the result of adaptive phenotypic plasticity. However, a comparison with results without plasticity (panel E) shows that this interpretation may be wrong.

Figure 6, left panels, illustrates that non‐symmetrical trait distributions result in non‐linear mean phenotype versus environment functions also in cases where the individual fitness peak position is a linear function of a dominating environmental factor. Figure 6, right panels, illustrates that other factors than the environment may contribute to non‐linearities in the mean phenotype versus environment function.

Panel 7, left panels, illustrates a case with slow adaptation as in, for example, Toljagic et al. (2018), where a mean trait value lags behind changes in the adaptive peak. The result is a non‐linear mean phenotype versus environment function, but also in this case mean phenotypic values over time can be predicted from a limited number of samples. Panel 7, right panels, shows that a narrow fitness function just as must be expected results in reduced mean fitness.

### 5.3 Discussion of the Real Data Case

The time series in the real data case in Section 4 has samples from around 2 million years ago and from around 0.5 million years. Although it would have been nice with additional data from around 1.4 and 0.25 million years ago, the available data indicate a linear adaptive peak versus environment function. This is especially clear in Figure 9C, based on moving average means of the $\partial^{18}O$ data. Considering the large standard errors in the *log AZ area mean* data it is in any case no clear indications of a very nonlinear function. It also seems reasonable to assume that long‐persisting species in the quite variable $\partial^{18}O$ environment in general have adaptive peak versus environment functions that are not too difficult to track, except for the last extreme environment, at least in the sense that the mean phenotypic value fluctuates around a moving average as in Figure 3A.

Note that the data from living or recent bryozoans are excluded from the analysis on the ground of extreme $\partial^{18}O$ values that do not fit into a linear model. These values are around zero (figure S9 in Liow et al. 2024), and thus much lower than the last value in the $\partial^{18}O$ time series in Figure 1. With the linear function in Figure 9C, this would give *log AZ area mean* values around 9.3, which is much lower than the values given in figure S2 in Liow et al. (2024). The reason for these discrepancies may be that the individual fitness peak versus environment function no longer is linear for extreme $\partial^{18}O$ values, or that the adaptive peak versus environment function is nonlinear owing to constraints as in Figure 6A–D.

The results in Figure 9 show that the mean phenotypic values can be predicted quite well with use of a linear adaptive peak versus environment function and moving average means of the $\partial^{18}O$ data with window size 100 samples, although the predictions for samples number 6 and 8 fall slightly outside of the error bars. With window size 120 only the prediction for sample number 8 falls outside of the error bar, but then the MSE value increases from 0.0016 to 0.0018. This indicates that each colony mean as used for computation of the population means in figure S6 in Liow et al. (2024) ideally should be weighted according to the $\partial^{18}O$ value for the colony. Also note that Figure 9 gives continuous predictions, although extensions to time periods long before the first and far beyond the last data point would be speculative.

The prediction results in Figure 9B, show that the optimal predictions with window size 100 have two nice properties. First, the rather rapid mean trait variations around 0.5 million years ago are followed pretty well, and second, the stabilization of the main trend from 0.8 million years ago and onwards are accounted for. When the window size is increased to 800, the first of these properties is lost, but the predictions are still clearly better than for random walk models (Table 3).

The prediction results using moving average means of the $\partial^{18}O$ data were validated by leave‐one‐out cross validation, with results included in Figure 10. Validation gave ${\mathrm{MSE}}_{\mathrm{CV}}=0.0020$, which should be compared with ${\mathrm{MSE}}_{\min}=0.0016$ for the predictions. As shown in Figure 10, the validation results are thus quite satisfactory in that all predictions of the left‐out samples except two fall within the error bars.

Use of mean $\partial^{18}O$ values for the different fossil formations worked less well (Figure 8), indicating that the colony means from each formation (raw data points as shown in Figure 2 in Liow et al. 2024) should, if possible, not all be considered as having the same $\partial^{18}O$ value. This underlines that there are not only phenotypic variations as indicated by the error bars in figure S2 in Liow et al. (2024), but there are also errors along the time axis.

It is of course an underlying assumption that the continuous mean trait predictions in Figures 9 and 10 not only give values that can be compared to mean values from collected data, but that also mean values in time periods without a known or investigated fossil record are predicted. That remains to be verified, and it would be especially interesting if the pronounced variations around 1.5 million years ago could be reproduced.

The AIC results in Section 4.3 show that the WLS model with use of moving average filtering of the $\partial^{18}O$ data clearly outperforms the URW model proposed in Liow et al. (2024). In addition to a much better AIC result, the WLS model also gives continuous predictions, while the URW model only gives a description that has to be interpreted. Since the WLS solution clearly points to directional selection, the URW result is also misleading. It is however interesting to note that the URW model gives a somewhat better AIC result than a WLS model based on mean $\partial^{18}O$ values for the different formations (time windows in Table 1). It is thus the moving average filtering of the $\partial^{18}O$ signal as in Figure 9 that makes the WLS model clearly better than the random walk models, but maybe the results in Figure 8 could become even better with more detailed information on the time points for the various colonies as indicated in Figure 2I in Liow et al. (2024)?

Liow et al. (2024) report that plasticity plays a role in the real data case, although in a complex way owing to the clonal and colonial nature of the data. There is, in fact, a theoretical possibility that the apparently linear mean phenotype versus environment function is caused by adaptive plasticity such that the mean phenotypic value tracks the fluctuations in sea temperature with small errors. As pointed out above, however, a linear mean phenotype versus environment function does not necessarily imply plasticity.

It is worth noting that just by looking at Figure 1 it might be tempting to approximate the mean phenotypic values as a linear function of the moving average of the environment. The results in Figures 9 and 10 could thus have been found by an *ad hoc* least squares approach, without any consideration of the theoretical background. However, the discussion of the tracking model in Figure 2 shows that the prediction results have a more general theoretical foundation.

As the WLS model in the real data case clearly outperforms random walk models, it is tempting to investigate if this is so also in similar cases where sea temperature may have been a dominant driver. The shell conicity as a function of time in Figure 7 in Hunt (2006) seems to be an interesting example, provided that good enough data over sea temperature in the Cretaceous period are available.

It is finally worth noting that the improved prediction results from the use of a WLS tracking model are in line with the main message in Pearl and MacKenzie (2018) that a causal model potentially performs better than a purely statistical model, provided that the cause is well known and understood. The prediction results in the real data case in Section 4 show that the cause of the changing mean phenotypic values is a well‐known dominating environmental driver. With large errors in the measurements of the driver, we would otherwise have had a serious errors‐in‐variables problem (Ergon 2025).

## Author Contributions

**Rolf Ergon:** conceptualization (equal), data curation (equal), formal analysis (equal), funding acquisition (equal), investigation (equal), methodology (equal), project administration (equal), resources (equal), software (equal), supervision (equal), validation (equal), visualization (equal), writing – original draft (equal), writing – review and editing (equal).

## Conflicts of Interest

The first author of Liow et al. (2024) which is a main reference, Lee Hsiang Liow, is my daughter‐in‐law. Otherwise, there are no conflicts of interest.

## Data Availability

MATLAB code and data are archived on bioRxiv https://doi.org/10.1101/2024.10.30.621046.

## References

[ece371705-bib-0001] Åström, K. J. , and R. M. Murray . 2008. Feedback Systems: An Introduction for Scientists and Engineers. Princeton University Press.

[ece371705-bib-0002] Banks, H. T. , and M. L. Joyner . 2017. “AIC Under the Framework of Least Squares Estimation.” Applied Mathematics Letters 74: 33–45. 10.1016/j.aml.2017.05.005.

[ece371705-bib-0003] Bell, M. A. 2013. “Adaptive Landscapes, Evolution, and the Fossil Record.” In The Adaptive Landscape in Evolutionary Biology, edited by E. Svensson and R. Calsbeek . Oxford University Press. 10.1093/acprof:oso/9780199595372.001.0001.

[ece371705-bib-0004] Di Martino, E. , and L. H. Liow . 2021. “Larger Offspring Associated With Lower Temperatures Across Species of Microporella, a Widespread Colonial Invertebrate.” Marine Ecology Progress Series 662: 1–13.

[ece371705-bib-0005] Ergon, R. 2025. “Errors‐In‐Variables and Validation Problems in Reaction Norm Predictions for Wild Populations.” Modeling, Identification and Control 46, no. 1: 13–33. 10.4173/mic.2025.1.2.

[ece371705-bib-0006] Estes, S. , and S. J. Arnold . 2007. “Resolving the Paradox of Stasis: Models With Stabilizing Selection Explain Evolutionary Divergence at All Timescales.” American Naturalist 169: 227–244. 10.1086/510633 17211806

[ece371705-bib-0007] Gingerich, P. D. 2001. “Rates of Evolution on the Time Scale of the Evolutionary Process.” Genetica 112, no. 113: 127–144. 11838762

[ece371705-bib-0008] Goodnight, C. J. 2013. “Wright's Shifting Balance Theory and Factors Affecting the Probability of Peak Shifts.” In The Adaptive Landscape in Evolutionary Biology, edited by E. Svensson and R. Calsbeek . Oxford University Press. 10.1093/acprof:oso/9780199595372.001.0001.

[ece371705-bib-0009] Hansen, T. F. 1997. “Stabilizing Selection and the Comparative Analysis of Adaptation.” Evolution 51: 1341–1351. 28568616 10.1111/j.1558-5646.1997.tb01457.x

[ece371705-bib-0010] Hunt, G. 2006. “Fitting and Comparing Models of Phyletic Evolution: Random Walks and Beyond.” Paleobiology 32: 578–601.

[ece371705-bib-0011] Johnson, R. A. , and D. W. Wichern . 2008. Applied Multivariate Statistical Analysis. Prentice‐Hall.

[ece371705-bib-0012] Lande, R. 1979. “Quantitative Genetic Analysis of Multivariate Evolution, Applied to Brain:Body Size Allometry.” Evolution 33: 402–416. 28568194 10.1111/j.1558-5646.1979.tb04694.x

[ece371705-bib-0013] Lande, R. 2009. “Adaptation to an Extraordinary Environment by Evolution of Phenotypic Plasticity and Genetic Assimilation.” Journal of Evolutionary Biology 22: 1435–1446. 10.1111/j.1420-9101.2009.01754.x. 19467134

[ece371705-bib-0014] Liow, L. H. , E. Di Martino , M. Krzeminska , M. H. Ramsfjell , S. Rust , and P. D. Taylor . 2017. “Relative Size Predicts Competitive Outcome Through 2 Million Years.” Ecology Letters 20, no. 8: 981–988. 10.1111/ele.12795. 28614907

[ece371705-bib-0015] Liow, L. H. , A. Porto , and E. Di Martino . 2024. “Trait‐Fitness Associations via Fecundity and Competition in a Two‐Million‐Year‐Long Fossil Record.” American Naturalist 204: 258–273. 10.1086/73133. 39179234

[ece371705-bib-0016] Lisiecki, L. E. , and M. E. Raymo . 2005. “A Pliocene–Pleistocene Stack of 57 Globally Distributed Benthic *δ* ^18^O Records.” Paleoceanography 20: A1003. 10.1029/2004PA001071.

[ece371705-bib-0017] Pearl, J. , and D. MacKenzie . 2018. The Book of Why. Basic Books.

[ece371705-bib-0018] Pigliucci, M. 2013. “Landscapes, Surfaces, and Morphospaces: What Are They Good for?” In The Adaptive Landscape in Evolutionary Biology, edited by E. Svensson and R. Calsbeek . Oxford University Press. 10.1093/acprof:oso/9780199595372.001.0001.

[ece371705-bib-0019] Toljagic, O. , K. L. Voje , M. Matschiner , L. H. Liow , and T. F. Hansen . 2018. “Millions of Years Behind: Slow Adaptation of Ruminants to Grasslands.” Systematic Biology 67, no. 1: 145–157. 10.1093/sysbio/syx059. 28637223

[ece371705-bib-0020] Uyeda, J. C. , T. F. Hansen , S. J. Arnold , and J. Pienaar . 2011. “The Million‐Year Wait for Macroevolutionary Bursts.” National Academy of Sciences of the United States of America 108: 15908–15913. 10.5061/dryad.7d580. PMC3179053 21873251

[ece371705-bib-0021] Voje, K. L. 2023. “Fitting and Evaluating Univariate and Multivariate Models of Within‐Lineage Evolution.” Paleobiology 49, no. 4: 747–764. 10.1017/pab.2023.1. 37859727 PMC7615219

[ece371705-bib-0022] Walsh, B. , and M. Lynch . 2018. Evolution and Selection of Quantitative Traits. Oxford University Press.

